# 

**Published:** 1915-07-03

**Authors:** 


### Presentation for War Service.

LIEUTENANT JOSEPH MCGRATH, M.B., has been presented by his friends in South London with a service sword and a case of surgical instruments. The presentation was under the auspices of the Villans Club, a social club, composed of medical practitioners, founded by Dr. A. E. Read, of Walworth. There was a large professional gathering at the function at the Manor Place Baths, Walworth, at which Lieutenant McGrath was congratulated on receiving his commission in the R.A.M.C. The gifts bore the simple inscription "To Lieutenant Joseph McGrath, M.B., our esteemed friend."

### Doctors' Wills.

DR. WILLIAM THOMAS EDWARDS, M.D., F.R.C.S., who died on April 11, aged ninety-three, has left estate valued at £80,955 gross, with net personalty £80,770. Among the bequests mentioned in his will are—£500 each to the Royal Medical College, Epsom, and the Medical Benevolent Fund, Chippenham; £1,000 and some instruments and books to Jones Hill Turner, for many years his assistant; £500 to his coachman; and £400 to his cook. In the event of the failure of the trusts on which the residue of his estate is left, the testator bequeathed £7,000 to the University College, South Wales and Monmouthshire, towards the maintenance of the Medical School; £5,000 to the Glamorganshire and Monmouthshire Infirmary; and £1,000 to the Blind Asylum or Workshop, Cardiff.

Rates of Subscription: Twelve months, 6/6; Six months, 4/-; Three months, 2/-, post free to any address in the United Kingdom. Abroad, Twelve months, 8/8; Six months, 5/-; Three months, 2/6, post free.—Address The Subscription Dept., "The Hospital," The Hospital Building, 28/29 Southampton Street, Strand, London, W.C.

